# An Evaluation of the Quality of Environmental Impact Assessment Reports in the Mobile Telecommunications Infrastructure Sector: The Case of Plateau State in Nigeria

**DOI:** 10.3390/ijerph191912659

**Published:** 2022-10-03

**Authors:** Vincent D. Choji, Isaac T. Rampedi, Lee-Ann S. Modley, Ayodeji P. Ifegbesan

**Affiliations:** 1 Department of Geography, Environmental Management & Energy Studies, University of Johannesburg, Johannesburg 2006, South Africa; 2 Department of Arts and Social Sciences Education, Olabisi Onabanjo University, Ago-Iwoye 120107, Nigeria

**Keywords:** environmental impact assessment, reports, mobile telecommunication infrastructure, Lee and Colley review package, satisfactory quality, unsatisfactory quality

## Abstract

Environmental impact assessment reports meant for proposed development actions can be evaluated to reveal their quality and fitness for the purpose of environmental decision-making. Therefore, this study evaluated the quality and identified strengths and weaknesses in environmental impact assessment reports of telecommunications infrastructure proposed for Plateau State in Nigeria. To this end, 80 reports were evaluated using the modified version of the Lee and Colley review package. The results revealed the following points. In Review Area 1.0 (Description of the proposed telecommunications facilities) and Review Area 5.0 (Communication of results), the quality of environmental impact assessment reports was found to be generally satisfactory. However, the quality of all reports was considered ‘very unsatisfactory’ (‘F’) regarding their overall legal compliance with the requirements stipulated in the remaining three Review Areas, namely, Review Area 2.0 (Terrain susceptibility in the proposed project areas), Review Area 3.0 (Associated and potential environmental impacts), and Review Area 4.0 (Mitigation measures/alternatives). This ‘F’ rating was assigned to 65% (52/80) of reports regarding Review Area 3.0 because the information provided was ‘very unsatisfactory’; important tasks were poorly carried out or not attempted at all. Moreover, in review areas such as Review Area 2.0 and Review Area 4.0, all reports in the evaluation were assigned an ‘F’ quality. Such an unsatisfactory quality rating is ascribable to the very unsatisfactory manner in which the reports were populated, especially as important task(s) were poorly performed or not attempted at all. Historically, only Review Area 1.0 and Review Area 5.0 indicated improvements in quality over time, whereas the remaining three review areas (Review Area 2.0, Review Area 3.0 and Review Area 4.0) did not improve. Based on the results obtained from the study, we recommend that there should be periodic reviews of environmental impact assessment reports by independent reviewers and environmental consultants should adhere to the sectoral guidelines for telecommunication infrastructure during the production of these reports. Moreover, in order to build technical capacity, more studies on report quality must be conducted in all sectors in Nigeria.

## 1. Introduction

The planning value of an environmental impact assessment (EIA) as a tool for determining and appraising the environmental feasibility of proposed development projects is widely recognised [1,2,3]. Since its inception in the United States, the EIA process comprises many steps, including the identification, prediction, evaluation, and mitigation of positive and negative impacts from proposed projects prior to their implementation [2,3,4]. An EIA is an important mechanism to describe the receiving environments, the assessment of alternatives associated with the proposed projects and their sites, and the selection of appropriate mitigation measures while interested and affected stakeholders are given an opportunity for involvement, consultation, and collaboration. Thus, the process brings sustainability considerations into the evaluation of proposed projects, the prime goal being to ensure that the receiving environments are not negatively affected and the threats to human health are avoided as much as possible. Despite the adoption of EIAs in many countries, research has shown that their effectiveness is constrained by several factors, especially in developing countries. According to Kamijo and Huang [5], EIAs in developing countries are constrained by many factors, such as the poor identification of anticipated impacts and the evaluation their magnitude and significance, as well as failure to design appropriate mitigation measures and involve affected stakeholders in a timely and meaningful manner. Even more problematic is the quality of EIA reports (EIARs), which give account of how the various stages of the process were managed and documented, thus raising questions on the completeness and comprehensiveness of such documents.

The inferior quality of EIARs in developing countries is one of the constraints that has affected national EIA systems over many years [6,7,8,9,10,11]. In many developing countries, the poor quality of EIA documentation has been attributed to inadequate organisational resources, lack of experience and relevant information on international best practices, as well as weaknesses around impact analyses and identification as well as mitigation planning [12,13,14]. Hence, the quality of EIARs is one of the key factors towards realising EIA effectiveness [5,11,14]. According to Caro and Toro [15], the quality of EIARs hinges on the extent to which they provide relevant information, not only to the interested and affected stakeholders, but also to regulatory authorities whose key responsibility is to ensure maximum enforcement and compliance with existing legal and regulatory requirements and international best practices.

In the evaluation of EIARs, regulators would like to ascertain that proposed projects are assessed from a life cycle perspective and the suggested mitigation measures are appropriate to the scale and significance of predicted environmental impacts, thus maximising positive impacts while preventing or reducing the intensity of negative impacts [3]. Thus, many studies have been conducted to review EIAR quality in developing countries such as Pakistan [8], Tanzania and Kenya [16], and Zimbabwe [17]. In addition, South Africa has witnessed a proliferation of research with mixed results on the quality of EIARs prepared for proposed projects in different sectors of the national economy [18,19,20]. For instance, a 2008 study conducted in South Africa on the quality of EIARs in the mining industry indicated that 85% out of the 20 reports assessed were of a satisfactory quality, and generally, they did comply with international best practices [21]. Sections of the reports that were based on the presentational and descriptive tasks were assigned comparatively higher quality grades than the tasks that required analyses of impact magnitude and the identification of project and site alternatives [21]. On the other hand, some studies found that the analytical review areas such as impact assessment actually performed relatively superior than it was the case with the descriptive sections in the EIARs compiled for the licensing of tourism projects in one of the biggest biosphere reserves in South Africa (i.e., the Kruger to Canyons Biosphere Reserve) [22]; thus, this differed from the results obtained by related studies in South Africa [23,24,25] and abroad [26,27,28]. 

The aforementioned studies show that the quality of EIARs is very important for informing various stakeholders who play an important role in the EIA process, especially on the key priority issues and significant impacts that require proper mitigation before projects can be authorised for construction and operation. Therefore, increasing our knowledge of EIAR quality is important because it can lead to the identification and analysis of strengths and weaknesses, which can provide key information towards improving EIA practice, thus enabling regulatory authorities to make informed and better decisions based on submitted EIA applications as to whether they are adequate and satisfactory for environmental authorisation [21,26]. Moreover, an EIAR is the final document during the EIA process; therefore, it should provide all the environmental information necessary for the impact assessment of proposed projects, thus informing joint decision-making in their sustainability considerations [3]. In view of the aforementioned rationale and justification for studies of this nature, this study was based on the evaluation of EIAR quality for proposed mobile telecommunications projects in the Plateau State of Nigeria; a country that has witnessed limited research on EIAR quality. Apart from the quality evaluation, the strengths and weaknesses in the reports were identified, consequently yielding important insights to the effectiveness of the EIA reporting processes in the study area. It was also imperative to determine whether the quality of EIARs was improving between the selected study periods, namely, Period 1 (between 2006 and 2012) and Period 2 (between 2014 and 2015). However, before the research methodology is explained, it is imperative to provide an overview of EIA legislation and regulation in Nigeria.

### 1.1. Overview of EIA Legislation and Regulation in Nigeria

The process of institutionalizing EIA in Nigeria dates to the year 1975. Their EIA process emerged from the establishment of the “Division of Urban Development and Environment” within the Federal Ministry of Economic Development [29,30,31]. However, in the beginning, more attention was focused on the environmental regulation of the impacts of the petroleum industry, because it was believed that this sector needed close environmental monitoring. At that time, most environmental legislation was in the form of pollution reduction measures that responded to local problems within the petroleum industry [30,31,32]. Consequently, many industrial regulations were established under the Petroleum Act (Act No. 51 of 1969) [33] to control the exploration of petroleum in Nigeria, and also to regulate the associated pollution aspects [34,35,36]. The application of EIA tools in all major projects of Nigeria was initiated in 1987 and was given a legal mandate in 1992 [30,31]. This was in response to the illegal dumping of about 3880 tons of hazardous toxic wastes, containing polychlorobiphenyls [37,38]. The guilty party in the dumping of such hazardous toxic wastes included the Italian vessel in the coastal town of Koko in Bendel State in 1987 [36,37,38]. The Federal Government of Nigeria responded by promulgating the Harmful Wastes Decree 42 of 1988 [36,37,38]. Embedded in this decree was a legal framework for the effective monitoring and control of the disposal of toxic and hazardous wastes in any part of Nigeria [38,39]. This decree also facilitated the establishment of the Federal Environmental Protection Agency (FEPA) through Decree 58 of 1988 [40]. The overall mandate of this Agency was to protect and develop precautionary environmental measures in Nigeria. In translating this mandate into action, a national policy called the Environmental Impact Assessment Act (Act No. 86 of 1992) was gazetted [36,37,40]. FEPA was then transferred and absorbed into the Federal Ministry of the Environment (FMENV). Subsequently, all States and Local Councils were required to establish environmental regulatory bodies to maintain environmental sustainability [40]. However, the focus of the state environmental protection agencies was on the collection of solid wastes and their disposal in the peri-urban areas of cities which are susceptible to the illegal dumping of wastes [41].

Given all these institutional and legal processes, Nigeria currently has three independent EIA regulations: the Petroleum Act of 1969, the Town and Country Planning Decree 88 of 1992, and the EIA Decree 86 of 1992 [30,39]. Inevitably, the development of each of these EIA systems in Nigeria is at a different stage of evolution [30,35,39]. The Petroleum Act and the EIA Decree have developed complex guidelines and legislation but are rated very low in terms of effective implementation and practice [12], while the Town and Country Planning Decree has not developed satisfactorily [30]. Moreover, the EIA Decree No. 86 of 1992 [30,39] has not been reviewed since its emergence, and the Federal Ministry of the Environment remains the only EIA regulatory authority that can conduct EIAs in Nigeria [30]. Therefore, attention concerning the development of key guidelines has been shifted to EIA Decree No. 86 of 1992 [42], with less interest towards the remaining two EIA systems.

The rationale for establishing the first EIA system in Nigeria was provided in the Petroleum Act (1969), which was aimed to address local environmental problems as well as reducing pollution in the petroleum industry [39]. Addressing issues relating to regional planning and development control for urbanization led to the enactment of the Town and Country Planning Decree of 1992 [43]. On the other hand, the EIA Decree 86 of 1992 aimed to cater to proposed development actions for most economic sectors at a national level [30]. The decree provided 19 major groups of such projects under the mandatory study activities, which is a list of projects that require compulsory EIA before authorization. The group of projects under the mandatory list for EIA in Nigeria include agriculture, airports, drainage and irrigation, land reclamation, forestry, housing, industry, infrastructure, and ports. Other types of projects in the mandatory schedule are mining, petroleum, power generation, transmission, quarries, railways, transportation, waste treatment and disposal, water supply, fisheries, resorts, and recreational developments [42]. Thus, Category 1 projects include all projects that must undergo full EIAs before they can be authorized for construction and implementation or operations. Some examples of project types under Category 1 include power generation, mining, quarries, petroleum, water supply, and waste management; Category 2 projects involve the same projects as in projects under Category 1, but such projects in Category 2 are smaller in size as compared with projects in Category 1. The decree also made it clear that projects in Category 2 can undergo full EIAs when they are due to be sited in environmentally sensitive locations; otherwise, no mandatory EIA is required before environmental clearance is issued. Examples of such projects under Category 2 include mini-hydro developments and any small-scale developments. Category 3 projects comprise institutional, health, family planning, nutritional, and educational action proposals, especially when physical buildings are involved.

In all of the aforementioned categories of proposed projects (i.e., Category 1, 2; and 3), projects involving telecommunication infrastructure, especially mobile base transceiver stations (BTSs), are not specifically mentioned in the EIA mandatory study activities in Decree No. 86 of 1992 [42]. However, these projects are listed under Category 2 in the Checklist for the category of ‘telecommunication facilities’ in the EIA Procedural-Guideline of 1995 [44]. Several federal ministries and government agencies have enacted laws and regulatory guidelines for their monitoring and evaluation, as shown in Table 1. In particular, the statutory requirements for the development and maintenance of telecommunications infrastructure in Nigeria are based on the (1) Guidelines on Technical Specifications for the Installations of Masts and Towers [45] and (2) National Environmental (Standards for telecommunications and broadcast facilities) regulations [46,47]. According to Okong and Ochela [48], these guidelines are applied to enforce precautionary measures for protecting human health, and the promotion of safety and the wellbeing of citizens living near mobile telecommunication base stations (i.e., BTSs). Regardless of these regulatory provisions, not much is known on how well these projects are being assessed in Nigeria and how they are accounted for in terms of the suitability of selected sites where they are constructed. Therefore, one way to gauge this aspect is by evaluating the quality of EIARs compiled for environmental authorization. Research on the quality of EIARs seeks to establish the degree of conformance or non-conformance with existing legal and regulatory provisions, procedural compliance, adherence to international best practices, and the completeness of information presented in such reports. Therefore, researching this kind of problem will shed light on the extent to which existing guidelines are being complied with, thus determining areas of legal and regulatory conformance or non-conformance, but also adherence to good practices and the incidence of malpractices [26,49,50].

Previous studies have expressed numerous shortfalls in Nigerian EIA practice, sometimes with negative implications for the preparation and compilation of EIARs. For instance, the problem of inadequate and weak public participation during the scoping phase of environmental assessments leads to the poorer documentation of environmental and socio-economic issues in the generated reports [30]. Consequently, such reports are criticized for being inaccurate, imprecise, and unclear [54]. The extent of this problem is increased by the point that public comments related to environmental assessments in Nigeria are needed only at the stage of reviewing [55]; a national practice that is contrary to international best practices which require public inputs in the early stages of the EIA process [3]. Additionally, the lack of experienced EIA consultants and approval authorities is another shortcoming which leads to the generation of EIARs with an unsatisfactory quality [30,55]. Other shortfalls are linked to the excessive bureaucracy and, sometimes, the duplication of duties between the Federal Environmental Protection Agency and the Federal Ministry of Petroleum Resources, thus negatively affecting set objectives of the EIA process [35,56]. These challenges not only undermine existing EIA laws, but also the attainment of long-term environmental sustainability. This study focused on the evaluation of EIAR quality for projects dealing with the construction of mobile telecommunications; therefore, it is necessary to summarise major developments in the relevant sector.

### 1.2. An Overview of the Global System of Mobile Communication (GSM) and Base Transceiver Stations (BTSs)

The global system of mobile telecommunication (GSM) was initially a European standard for telephony and was introduced in 1992 [57]. However, it is now widely used around the globe [48]. Since its development in 1992, the telecommunication industry has achieved rapid growth in all countries [58]. For example, while the world population was estimated to be nearly 7.676 billion around 2019 [59], the total number of mobile subscribers grew from 2.5 billion in 2006 [60] to 5.112 billion in 2019 [59]. Moreover, in 2018, the industry provided 14 million jobs with an additional 17 million indirect jobs globally [58]. The increased adoption of GSM technologies arises from their relevance and enhancement of human activities such as in the spheres of transportation, agriculture, banking, security, disaster risk management, and health care services, amongst others. While recognising the positive economic and social impacts associated with the GSM and its BTS infrastructure, a number of negative impacts have been identified, including declines in bee populations [61,62], the development of cancer cells in people and animals, and many other health challenges which have emerged [63].

Nigeria adopted the use of GSM technology in 1999 when the country completed a de-regularisation exercise on the telecommunication sector which had started as early as 1992 [64,65]. It was in August 2001 that the first call using the GSM technology was made in Nigeria, and this completely changed the manner in which the telecommunication sector was managed and operated [66]. By May 1999, there were only 450,000 telephone lines for an approximate population of 120 million people, but during the 1999–2007 period, the number of subscribers had risen to 38 million [64,65]. In addition, by the end of 2018, the number of GSM subscribers had once again increased to 174 million [67]. Furthermore, it is estimated that there are 298 million GSM subscribers in Nigeria who are served by 53,000 BTSs. Moreover, the contribution of the telecommunication industry to the national economy has witnessed continuous growth, ranging from 7.40% in 2013 to 10.11% in 2019 [68]. However, this accelerated development in the telecommunication sector of Nigeria has attracted the demand for and use of products and accessories such as mobile phones, iPads, and computers [69]. As a result, large quantities of electronic waste (1.1 million tons) are generated annually in the country [70,71]. Finally, some studies have confirmed health challenges such as cancer, headaches, fatigue, dizziness, sleep disorders, memory loss, skin itching, anxiety, and poor eyesight in people living close to BTS infrastructure [71,72,73].

## 2. Methodology

### 2.1. The Lee and Colley Review Package and Its Modification

**Figure 1:**
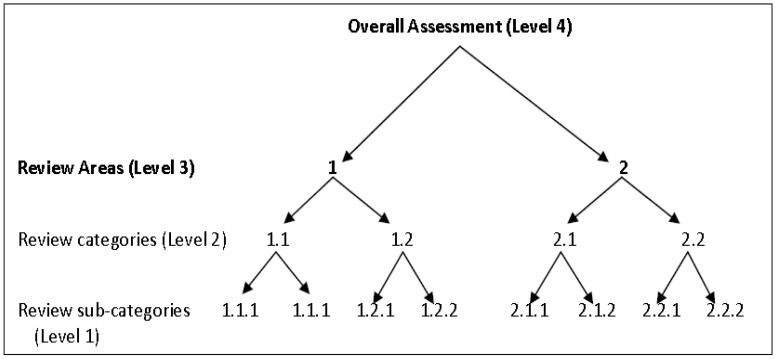
The original hierarchical Lee and Colley review package, but showing only two review areas, review categories, and review sub-categories. (Adapted from Lee et al. [74]).

The Lee and Colley review package has been used internationally to evaluate the quality of EIARs [74,75,76,77]. This package consists of several criteria for assessing the quality and the standard at which specific environmental assessment tasks undertaken during the EIA process are being documented in the EIARs. The Lee and Colley Review Package was initially only used for EIAR quality assessment in the United Kingdom, but it has since been widely adapted to suit different legal and regulatory requirements in many countries due to its flexibility [3]. The package consists of a hierarchically structured evaluation framework (Figure 1) [3,27].

The original version of this framework had only four review areas (RAs), and under each area are relevant review categories (RCs), and review sub-categories (RSCs) (Figure 1). However, in Figure 1, only two review areas are indicated for illustration purposes, instead of showing all of them. In making use of this framework, the process of EIAR quality review begins with the evaluation of review sub-categories (RSCs) that are located at the base of the hierarchy. Then, the reviewing process progresses to the next layer of the hierarchy, which comprises review categories (RCs); thirdly, the same evaluation exercise is then extended to the broader review areas. The quality for each environmental assessment task in each level of the hierarchy (Figure 1) is determined by assigning specific symbols or grades to the various EIARs under examination, ranging from “A” to “F” levels (Table 2) [66,74].

The assigned quality symbols for each environmental assessment task in the EIARs are subsequently recorded in a collation sheet. The sheet is then used to decide on the quality to be assigned to each EIAR based on tasks that were performed satisfactorily or unsatisfactorily. In line with the approach adopted by previous studies [76,77], the assignment of satisfactory quality to EIARs in the present sample was based on the allocation of symbols such as “A”, “B”, or “C” (Table 2). Degrees of satisfactory quality ranged from the “A” (generally well performed and very satisfactory) symbol, to “B” (generally satisfactory and complete), to “C” (just satisfactory despite omissions or inadequacies). In this continuum, the symbol “C” represented the lower limit and borderline of satisfactory quality. Furthermore, allocating these symbols depends on the extent to which individual EIARs complied with certain criteria. For instance, in the present study, for EIARs to be regarded as having satisfactory quality, they had to comply with the following requirements and best practices recognized in the literature [28,78].

- All the legal, regulatory, and procedural requirements must be met;
- Description and assessment of environmental sensitivities are needed;
- Accurate identification and prediction of impacts must take place;
- Sound mitigation planning throughout the project’s life cycles is needed;
- Sufficient description of proposed infrastructure, project sites, and associated environmental baselines must be provided;
- Adequate public participation for joint environmental decision-making is needed;
- Proper identification and analyses of alternatives are required;
- Proper communication of EIA results is mandatory.

### 2.2. Structure of the Adapted Lee and Colley Review Package

For the evaluation of the quality of EIARs in the present study, the Lee and Colley review package was selected. However, it was modified to suit the context of the EIA process and industry requirements for the telecommunications sector of Nigeria. Some of the requirements are specified in the National Environmental Standards for Telecommunications and Broadcast Facilities in Nigeria [46]. Furthermore, other criteria were obtained from the World Bank guidelines for telecommunications infrastructure [79], which are an indication of international best practices in the writing of EIARs. In addition, the EIA guidelines for the review of EIA reports in Nigeria [47] recommend, amongst others, the use of Lee and Colley review criteria for EIAR quality evaluation

As already indicated, the results on the EIAR were recorded in a collation sheet. The sheet is used to allocate symbols per the quality of specific environmental assessment tasks undertaken. The best-performed EIARs will be those whose quality ranges from “A-B”, whereas the “E-F” category represents poorly written EIARs. In determining how satisfactory or unsatisfactory various environmental assessment tasks at each hierarchical level were, it is important to emphasize that grades for higher levels of the hierarchy were not determined on the basis of numerical accounting or aggregation, but by qualitatively considering the overall performance grade per each category, and the same applies for review areas [7,20].

As stated earlier, the original Lee and Colley review package had only four review areas, entailed: (1) description of the proposed development, the local environment, and baseline conditions; (2) identification and evaluation of environmental impacts; (3) alternatives and mitigation of impacts; and (4) the communication of results [74]. However, in the current study, the original Lee and Colley package was modified to be in line with the Nigerian EIA legal and regulatory context. As a result, the total number of review areas (RAs) amounted to five instead of the original four; a summary is provided in Table 3. Furthermore, the number of corresponding review categories (RCs) became 26, whereas the smaller review sub-categories (RSCs) were 158 in number. This modification of changing the number of review areas in the original Lee and Colley review package has also been successfully performed in related studies. For example, Aung et al. [13] and Aniwofose et al. [76] used five different review areas instead of the traditional four in the original Lee and Colley review package.

Given the information shown in Table 3, it is important to give a detailed account of how the original Lee and Colley review package was modified, especially in terms of review categories (RCs) (Table 3) and review sub-categories (RSCs). In this study, the additional RCs that were introduced in the modified Lee and Colley review package were derived from three regulatory guidelines namely, the (1) Guidelines on Technical Specifications for the Installations of Masts and Towers; (2) National Environmental Standards for Telecommunications and Broadcast Facilities; and (3) Guidelines for the Review of EIARs in Nigeria [45,46,47]. The EIA guidelines for the commissioning of telecommunications infrastructure in Nigeria are derived from the World Bank EIA guidelines for telecommunication infrastructure [80], which represents international best practices. All the resulting RAs and RCs are shown in Table 3, and conformance with them can be regarded as an indication of quality not only in the process of impact assessment, but also in the EIA reporting phase where major decisions for granting environmental licenses are undertaken. Similarly to the approach conducted in other EIA quality studies [27,75,76], the quality recorded for all review subcategories (RSCs) determined the quality of review categories (RCs), which, in turn, informed the quality of review areas (RAs), and finally, the overall quality of specific EIARs.

### 2.3. Collection and Selection of EIARs for Data Analyses

Following a formal request to access EIA documents, 288 EIARs were made available for the current study by the regulatory authority for EIA in Nigeria, which is the Federal Ministry of Environment (FMENV) in Abuja. These EIARs were made available on the condition of anonymity; hence, the details of the various project sponsors are not given in this study. All the selected EIARs were previously submitted to the regulatory government authorities between the years of 2006 and 2015 as part of applications for the environmental permitting of projects involving the establishment of GSM and BTS (mobile) telecommunications infrastructure.

Out of the 288 available EIARs compiled and reviewed specifically for the construction and maintenance of proposed telecommunications masts in Plateau State (Nigeria), the study grouped them firstly according to their year of environmental authorization, thus subjecting them to a stratified sampling process based on the following years: 2006; 2012; 2014; and 2015. As shown in Table 4, the numbers of EIARs applicable to 2006, 2012, 2014, and 2015 were not equal, thus posing a limitation in the selection of reports for the present quality appraisal. It must also be indicated that before 2006, there was only one EIAR compiled for the construction of telecommunications infrastructure in Plateau State. Thus, EIARs produced before 2006 were not sufficient to warrant an objective and comparative quality evaluation; hence, the research focused only on the periods ranging from 2006 to 2015. Further sampling was performed randomly; thus, 20 EIARs were chosen for each specific year.

Beyond the random sampling of 20 EIARs for the years 2006, 2012, 2014, and 2015, it was necessary to set apart at least two main review periods because one of the research objectives was to determine whether EIAR quality was improving or declining through these years. The review period simply means the time frame wherein different EIARs are grouped together to facilitate inter-period comparisons. However, setting the two review periods brought further constraints to the sampling procedures because the time frames were not equal in terms of the number of years: whereas Period 1 ranged from 2006 to 2012 (*n* = 7 years), Period 2 was relatively shorter (i.e., from 2014 to 2015 (*n* = 2 years). This is because, in certain years, fewer EIARs were submitted for approval, especially during 2006. Despite this inherent inequality, it was still possible to create at least two equal classes of data for comparative purposes.

In the assignment of quality for every EIAR that is reviewed in studies of this nature [13,14,74], a double review mechanism is always recommended even though it is not entirely possible to eliminate subjective impressions. In keeping with this principle, there were two independent reviewers for the assessments of EIAR quality. Where variations occurred in the allocation of quality symbols, the reviewers conducted the assessments jointly and then reached a consensus as to what should be the final quality for each EIA task under scrutiny. By making use of this mechanism, the degree of subjectivity in the allocation of quality symbols was significantly reduced while consistency in the grading of EIARs was enhanced.

Lastly, the determination of strengths and weaknesses amongst the selected EIARs was performed according to the method described in the relevant literature. Thus, we followed the approach previously executed by both Sandham et al. [18] and Sandham et al. [21] in South Africa for EIAR quality evaluation. In their research, areas of strengths and weaknesses were determined by calculating the proportions of EIARs that were classified or assigned into ‘A-B’ and ‘E-F’ quality grades for all review areas and associated review categories. In this manner, the review categories that received a percentage of ‘A-B’ grades that exceeded 50% were regarded as areas of strengths, while the percentage of grades that received ‘E-F’ grades of more than 50% were deemed to be areas of weaknesses. It was also possible to track improvements or decreases in EIAR quality between the two selected study periods.

## 3. Results

The results that came out of the data collection, processing, analysis, and interpretation are presented for the different review areas, review categories, as well as review subcategories. This is followed by the identification of strengths and weaknesses in the sampled EIARs. Lastly, changes in the quality of EIARs between the selected study periods are explained.

### 3.1. Overall Quality of Selected EIARS

**Figure 2:**
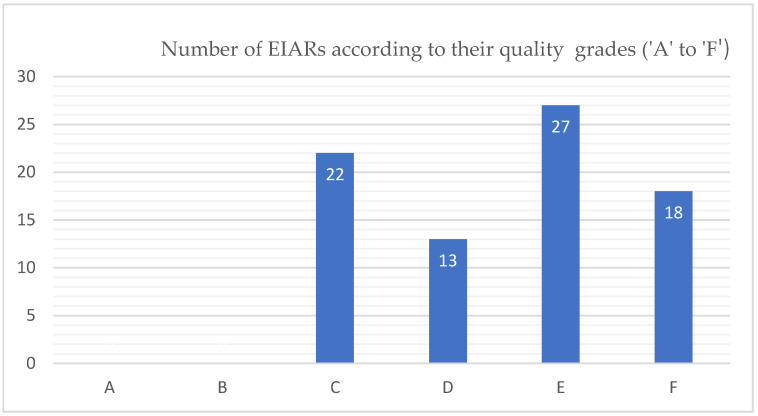
The numbers of EIARs according to the quality grades allocated to them.

As stated above, 80 EIARs were selected for the quality review. Figure 2 indicates that the majority (i.e., *n* = 58, 73%) of EIARs were found to be in the D-F quality class, meaning that they were grossly unsatisfactory due to significant omissions and inadequacies in their compilation and content. In terms of individual quality symbols, they were distributed as follows: 13 EIARs were assigned a ‘D’ symbol, 27 ‘E’, and 18 ‘F’. On the other hand, only 27% (*n* = 22) of EIARs were in the A-C quality category, with none assigned to the ‘A’ and ‘B’ grades’ (Figure 2).

### 3.2. Quality of Review Areas

**Figure 3:**
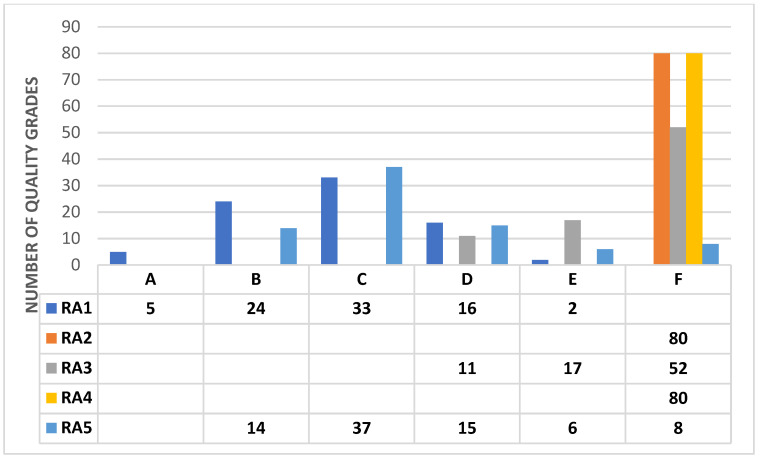
Quality scores or grades for different review areas. Key/Legend: RA1 = Descriptions; RA2 = Terrain susceptibility; RA3 = Impact predictions; RA4 = Mitigation/Alternatives; RA5 = Communication of results.

Figure 3 indicates the number of EIARs based on the quality symbols or grades allocated for their different review areas. With few exceptions, most review areas were assigned very poor reporting quality. Only two review areas, namely, RA 1.0 (Description of proposed telecommunications facilities and project sites) and RA 5.0 (Communications of results) exhibited an EIAR quality that was generally in the satisfactory (A-C) range (Figure 3). This is because important tasks were performed relatively adequately, although there were few omissions. In terms of meeting the requirements for RA 2.0 (Terrain susceptibility) and RA 4.0 (Mitigation and Alternatives), all EIARs were assigned an ‘F’ quality rating (Figure 3). This means that their quality was very unsatisfactory because important tasks were poorly carried out or not attempted at all. Lastly, all EIARs were assigned to the D-F class because they failed to satisfactorily meet the requirements for RA 3.0 (Impact identification and predictions).

#### 3.2.1. Review Area 1 (Description of the Projects and Environment)

Review Area 1 was based on the ‘Description of planned facilities and project sites’, which entailed proposed BTS infrastructure for mobile telecommunications in Plateau State (Table 5). All EIARs were populated satisfactorily (A-C) in terms of the requirements for this review area, although some had some omissions and inadequacies. The review area had eight categories, namely, ‘Planning arrangements’ (RC 1.1); ‘Spatial information relevant for the projects’ (RC 1.2); ‘Expected raw materials’ (RC 1.3); ‘Site construction activities in the base stations’ (RC 1.4); Site selection consideration (RC 1.5); ‘Baseline data’ (RC 1.6); ‘Description of the proposed project sites’ (RC 1.7); and ‘Potentials of the area’ (RC 1.8) (Table 5). It was found that 89% of EIARs were assigned satisfactory quality ratings in the A-C class in terms of meeting the requirements for RC 1.1 (Planning considerations), whereas only a few (6%) were allocated very unsatisfactory (E-F) quality ratings. Other review categories that complied in a satisfactory manner (A-C) included RC 1.4 (Site construction activities), RC 1.5 (Site selection considerations), and RC 1.6 (Environmental base lines).

The proportions of EIARs that were assigned to the A, B, and C classes were 75%, 78%, and 70%, respectively. Similarly, the relevant spatial information that provided geographical details in terms of specifying the specific names of chosen localities, topographical maps, and the GPS coordinates (RC 1.2) where the proposed facilities would be constructed were also written in a satisfactory manner, with 70% of EIARs falling in the A-C quality range. However, when it came to providing descriptions of the potential land uses of the selected areas (RC 1.8), the level of quality decreased markedly, with only 20% of EIARs assigned to the A-C class.

#### 3.2.2. Review Area 2—Terrain Susceptibility in the Proposed Project Areas

This review area had two review categories, namely, ‘Potential conflict/s with other land uses’ (RC 2.1) and the ‘Anticipated environmental problems in the project sites’ (RC 2.2) (Table 6). Overall, the requirements for these review categories were met in a very unsatisfactory manner. All (100%) EIARs were allocated to the E-F quality range regarding the requirements of RC 2.1. There was no attempt to provide any information needed to assess the relevant land use types nor their potential capabilities and terrain susceptibility in the proposed project sites. Although a few (n = 20; 25%) EIARs supplied satisfactory information (A-C) in terms of outlining some of the significant environmental problems prevailing or expected in the selected sites (RC2.2), the majority (75%) of them were found to be very unsatisfactory (E-F), with inadequate detail and uncompleted sections.

#### 3.2.3. Review Area 3—Impact Identification and Prediction

Review Area 3 was based on the identification and prediction of anticipated environmental impacts that would result from the proposed mobile telecommunications infrastructure. The results are shown in Table 7. This review area comprised six review categories, namely, ‘Terrestrial habitat alterations’ (RC 3.1), ‘Avian collisions’ (RC 3.2), ‘Aquatic habitat alteration’ (RC 3.3), ‘Visual impacts’ (RC 3.4), ‘Impact prediction’ (RC 3.5), and ‘Scoping’ (RC 3.6). The first four review categories were taken directly from the guidelines regarding the identification and assessment of impacts associated with the construction of mobile telecommunications infrastructure in Nigeria.

As shown in Table 7, generally no EIARs were able to identify and evaluate anticipated environmental impacts in a satisfactory manner even if some attempts were made. Inevitably, the quality of EIARs ranged from a ‘D’ symbol to the ‘F’ rating. The ‘D’ symbol was assigned mainly because certain parts were attempted although there were still gross inadequacies evident in 16 EIARs in terms of Terrestrial habitat alterations (RC 3.1) and 18 EIARs regarding anticipated Avian collisions with cell phone masts (RC 3.2). The identifications of both Visual impacts (RC 3.3) and Aquatic habitat alterations (RC3.4) were not satisfactorily (F) conducted, because important tasks were poorly performed or not attempted at all. Similarly, the information given on the Scoping processes (RC3.5) conducted was assigned an ‘F’ symbol in all EIARs as their quality was very unsatisfactory. Although the aforementioned impacts (i.e., habitat alterations (RC 3.1), avian collisions (RC 3.2), and visual intrusions (RC 3.3)) were poorly addressed, further data analysis revealed better impact predictions (RC 3.5) when it came to the treatment of other types of environmental impacts. Such impacts included:

- Socio-economic impacts;
- Loss of communal land parcels;
- Loss of biodiversity;
- Potential soil pollution;
- Water pollution;
- Noise pollution;
- Impacts on human health and safety.

#### 3.2.4. Review Area 4—Mitigation Measures/Alternatives

This review area consisted of five different RSCs: ‘Measures to reduce or avoid animals in all phases of the projects’ (RC 4.1), ‘Procedures to minimize avian collisions’ (RC 4.2), ‘Measures to prevent and control visual impacts’ (RC 4.3), ‘Hazardous materials management action’ (RC 4.4), and ‘Measures to minimize EMF radiation on biodiversity and man’ (RC 4.5). When looking at this review category as a whole (Table 8), all the review categories concerned were given very unsatisfactory quality ratings in the D-F class for all EIARs involved. This is one of the worst and poorly performed RCs based on the scores allocated to the corresponding RSCs under it, thus highlighting several shortcomings in the selected EIARs.

#### 3.2.5. Review Area 5—Communication of Results

To assess this review area, the most important aspects included the provision of a ‘Report Layout’ (RC 5.1), ‘Presentation style’ (RC 5.2), ‘Emphasis’ (RC 5.3), and the ‘Non-Technical Summaries’ (RC 5.4). Generally, it was found that the requirements for the review areas were met in a very satisfactory manner: 51 (64%) of the EIARs were deemed to be in the A-C quality category (Table 9). Similarly, when it came to EIAR report layout, 66 (83%) EIARs exhibited quality in the A-C range, while the requirements for the ‘presentation’ review category were carried out successfully (A-B) in 74 (93%) EIARs. Emphasising the relevant parts of the EIARs was also judged to be generally satisfactory (A-C) in 67 (83%) EIARS. However, all EIARs failed to satisfy the requirements for the proper writing of ‘Non-Technical Summaries’. As a result, such EIARs were assigned an ‘F’ quality symbol, meaning that this task was conducted very poorly; however, in the majority of cases, such summaries were missing.

### 3.3. Determining Areas of Strengths and Weaknesses

Based on the results obtained for all Review Areas (Table 5, Table 6, Table 7, Table 8 and Table 9), there are only two areas of strengths, namely, Review Category (RC) 1.1 (Planning) and Review Category (RC) 1.5 (Site selection considerations). The results are shown in Table 10. Nonetheless, under Review Category (RC) 1.1, twelve (11%) EIARs were assigned quality in the D-E class as they had inadequacies. In the same vein, 22% of EIARs were in the D-F quality category because they failed to meet the requirements in RC 1.5 in a satisfactory manner.

In contrast to the strengths indicated in Table 10, the research identified 13 areas which can be regarded as weaknesses in the compilation and composition of the various EIARs that were subjected to a quality review. As shown in Table 11, most of the weaknesses were found in RA 3.0 (Impact identification and assessments) and RA 4.0 (Mitigation/Alternatives).

### 3.4. Changes in the Quality of EIARs between Two Periods

Furthermore, in Table 12, a comparison in quality is presented between EIARs that were compiled and approved during the 2006–2012 period (Period 1) against those that were approved during the 2014–2015 period (Period 2). The biggest improvement in the quality of the EIARs occurred in RA 1.0 (Descriptions of projects and environments); the proportions of EIARs that were grouped under the A-C class improved markedly from nearly 63% during the 2006–2012 period to 93% in the 2014–2015 period. Similarly, those that were classed in the A-B group increased from 25% in the first period to 47.5% in the second period. The review categories under RA 1.0 that improved between the two study periods included: (1) RC 1.1 (Planning), RC 1.2 (Spatial information), and RC 1.7 (Description of project sites).

However, there were also instances where quality remained the same in both periods, notably for review categories such as RC 1.2 (Spatial information), RC 1.3 (Raw materials), and RC 1.4 (Site constructions). In terms of meeting the requirements for RA 5.0 (Communication of results), the proportions of EIARs in the A-C class increased from 63% to 65%. Similarly, the proportions of EIARs under RC 5.1 (Layout) and RC 5.3 (Emphases) (A-C class) rose from 80% to 85% and nearly 83% to 85%, respectively. Nevertheless, the quality of EIARs did not improve in RC 5.4 (Non-technical summary); meanwhile, minimum improvements were found in RC 5.2 (Presentation).

In addition, no major improvement in quality occurred in all EIARs in terms of meeting the requirements for RA 2.0 (Terrain susceptibility) as well as the two review categories that were grouped under it—RC 2.1 (Potential conflict with land uses) and RC 2.2 (Significant environmental problems in the project sites). The same pattern was also found in RA 4.0 (Mitigations/Alternatives), because there were no improvements in quality. Although the quality of EIARs never improved under RA 3.0 (Impact identification and prediction) and the underlying review categories, satisfying the requirements of RC 3.5 (Impact prediction) witnessed a slight improvement between the two study periods. This is because the proportions of EIARs that were allocated quality symbols in the A-C class increased from 73% (2006–2012 period) to 78% (2014–2015 period), respectively. Additionally, nearly the same improvement occurred for the proportions of EIARs that were assigned to the A-B quality range.

## 4. Discussion

The results derived from the present research indicated the quality of EIARs prepared for projects involving the development of mobile telecommunications infrastructure in the different areas of Plateau State (Nigeria). This was carried out by paying attention to five different review areas and their associated review categories and sub-categories. Based on the results generated by the study, only two review areas, namely, RA 1.0 (Description of facilities and project sites) and RA 5.0 (Communication of results), were found to be generally in line with the legislative and regulatory requirements for environmental assessments and best EIA practices for the development of telecommunication infrastructure in Nigeria. The remaining three review categories (RA 2.0, RA 3.0, and RA 4.0) were grossly unsatisfactory in terms of their quality. In providing an overview on the EIA system in Nigeria, an earlier study found that the quality of EIARs is very poor and is reflective of limited environmental assessment skills in this country, as well as the low competence of EIA consultants who compile such reports and the regulatory authorities who approve them [30]. What follows is a discussion of the findings stemming from the present research, starting with RA 1.0 (Description of facilities and project sites) and RA 5.0 (Communication of results). Thereafter, others review areas are given attention as well.

Providing an adequate description of planned development actions and associated project sites is important for all stakeholders involved in the EIA process in order to understand their main characteristics and the different activities planned for their life cycle [2,3]. This task is also imperative especially for countries that follow a list-based approach in deciding which projects require an EIA process before their commencement and construction. The results obtained for the description of proposed facilities in the present research revealed that 89% of EIARs were of satisfactory quality (A-C) ratings in addressing the requirements for RC 1.1 (Planning considerations), whereas only a few (6%) were allocated very unsatisfactory (E-F) quality ratings. Other review categories that featured predominantly in the A-C class involved RC 1.4 (Site construction activities), RC 1.5 (Site selection considerations), and RC 1.6 (Environmental base lines). These results are similar to the findings obtained not only in Nigeria, but elsewhere. For instance, in an EIAR quality review conducted for projects in the oil and gas sector in Nigeria, 90% of project activity descriptions were of a satisfactory (A-C) quality [76]. In addition, several studies in South Africa have reported similar findings attesting to the satisfactory descriptions of proposed activities in their selected EIARs [20,21,23]. Although this review area (RA 1.0) appeared to be of good quality at the review area level in the present research, it is important to highlight some of the quality deficiencies that were found in some of the review categories as well as review subcategories. For example, certain descriptions of project sites were incomplete. Hence, 65% of associated EIARs were assigned to the D-F class, meaning that they were unsatisfactorily populated by EIA consultants. Moreover, many EIARs were assigned an ‘F ‘quality level because they failed to provide meaningful descriptions on the following review subcategories:

- Review Sub-Category (RSC) 1.7.5 (Recreational areas): 57 EIARs (F-symbol);
- Review Sub-Category (RSC) 1.7.9 (Nature conservation areas: 67 EIARs (F-symbol);
- Review Sub-Category (RSC) 1.7.11 (Important agricultural /forestry areas: 44 EIARs (F-symbol).

Overall, the Review Area (RA 5.0) based on the ‘Communication of results’ was performed satisfactorily because approximately 60% of EIARs were assigned to the A-C quality class. However, the greatest area of deficient EIAR quality was seen in the compilation of their non-technical summaries. According to de Jesus [81], such summaries in an EIAR play a very important role for various stakeholder groups and competent authorities because they provide an easy-to-read and non-technical explanations of critical information, thereby assisting and enhancing meaningful public participation in the environmental assessment process. Despite such roles, in the present study, some of the summaries were completely missing, and where they were presented, they were not only too lengthy to be read conveniently fast, but they had unnecessary technical jargon or terms and abbreviations that were not explained further. In this way, the value of the summaries provided was substantially reduced, thus rendering them unhelpful for enhancing public understanding of the main EIA findings, as well as project descriptions, relevant alternatives, and proposed mitigation strategies.

The quality of EIARs regarding RA 2.0 (Terrain susceptibility in the proposed project areas) was found to be grossly deficient, because the relevant information was either inadequately given or totally missing. All EIARs failed (E-F) to meet criteria for RC 2.1 (Potential conflict with existing land uses). They were poorly and unsatisfactorily written in terms of assessing potential land use clashes and possible conflicts. Such inferior quality means that the EIA processes undertaken were limited in assessing the extent to which the proposed mobile telecommunications infrastructure projects were compatible or incompatible with existing land uses in the selected development sites. These weaknesses also undermine baseline terrain evaluations for the proposed projects and the identification of environmental interactions and impacts [82]. Moreover, delicate mobile telecommunication infrastructure is susceptible to damage by climatic elements, and even theft by local inhabitants or outsiders, especially in areas where civil protests happen intermittently, thus raising the need to have a good understanding of the prevailing socio-economic conditions in the selected development sites. For example, it has been estimated that in 2012 alone, 530 cell phone base stations were completely vandalized during social upheavals in Nigeria, with another 380 of them being destroyed by floods, while 150 base stations were damaged by the terrorist organisation known as Boko Haram [83]. Therefore, it is important to understand the various terrain sensitivities in the location of vulnerable infrastructure, thus avoiding potentially dangerous locations for them.

At the heart of the EIA process is the identification and assessment of anticipated environmental impacts [3]. With such knowledge, the process can assess the magnitude and significance of such impacts, as well as the extent to which they can be mitigated successfully. However, most EIARs in the present study exhibited very deficient quality (with 100% in the D-F class) ratings regarding the criteria set for RA 3.0 (Impact identification and assessment). This problem appears to be long-standing in Nigeria as previous researches have indicated the inadequate identification and description of probable environmental effects in some of the EIARs prepared for projects in the petroleum sector [40,84]. The deficient EIAR quality found in the present study is not in line with the EIA guidelines for the telecommunications sector in Nigeria, especially given the negative human health impacts associated with the mobile telecommunications infrastructure [71,72,85]. In addition, the present research found that key information concerning the different impacts (such as avian collisions, terrestrial habitat alterations, aquatic habitat alterations, and visual intrusions, amongst others) likely to be caused by cell phone base stations were not satisfactorily provided, thus contrary to good EIA practices in environmental assessment reporting, as highlighted in some of the international literature [3,11,86,87]. Furthermore, the present findings are in stark contrast with the patterns seen in the results generated by Anifowose et al. [76] for oil and gas projects in Nigeria, where up to 74% (i.e., 14) of their EIARs exhibited generally satisfactory (A-C) quality. Regardless of the low quality of impact identification and assessment of key impacts (i.e., avian collisions, terrestrial habitat alterations, aquatic habitat alterations, and visual intrusions, amongst others) in the present research, it is imperative to indicate that the impact assessment methodologies were provided generically and they were based on the traditional Leopold matrix method of impact prediction, the relevant formula used for this purpose, and applicable thresholds for low, moderate, and high impacts. In that context, 75% of the impact assessments performed were apportioned to the A-C quality class. Although such impact predictions are not necessarily out of place for the environmental assessment of mobile telecommunication infrastructure projects, it could have been more meaningful to distinguish between direct and indirect impacts; significant and insignificant impacts; cumulative and residual impacts during the scoping process, thus focusing more time and attention on the most significant impacts.

As specified earlier, Review Area 4.0 was based on the planned mitigation measures and the consideration of alternatives for the proposed mobile telecommunications projects in Plateau State. Project developers are obliged to specify, investigate, and analyse the most feasible mitigation measures, the prime purpose being to minimise and reduce the magnitude and severity of anticipated adverse environmental impacts during the life cycle of proposed development actions [2,18,88]. However, in the present study, these requirements came out to be one of the worst performing (i.e., D-F ratings) review area, judging from the multiple weaknesses that were identified in the selected EIARs. Furthermore, the consideration and evaluation of impacts associated with various alternatives is an important section in an EIAR and is tantamount to good EIA practice. In contrast, in the study carried out by Sandham et al. [21] in South Africa, it has been reported that the mitigation arrangements provided and the consideration of alternatives both exhibited relatively better quality because about 65% of their EIARs were assigned a ‘C’ quality rating or higher, although it was also conceded that there were inherent challenges. Furthermore, the Dutch EIA procedure (which is an example of an international best practice) makes the development of alternatives for proposed projects and the assessments of impacts associated with each alternative a mandatory task [89]. Similarly, Kamijo and Huang [90] (p. 144) maintain that the “identification and comparison of alternatives are central to the application of EIA as a creative, problem-solving process”. Therefore, one way to improve the quality of EIARs in developing countries, including Nigeria, is to pay attention to processes that involve the analysis of alternatives and public involvement and the linkages between them.

After providing a discussion of quality in the different review areas and associated review sub-categories and performing comparisons with the relevant literature, it is also important to reflect on the improvements in EIAR quality between the two study periods (2006–2012 and 2014–2015) (Table 12). Judging from the patterns summarised in Table 12, it is clear that there were only limited improvements in the quality of EIARs sampled for the two study periods. Moreover, in some instances, the quality of certain review areas and associated tasks never changed, whereas in some of the review areas, there were actually decreases in EIAR quality.

Nonetheless, key improvements were found in the review areas that required the performance of descriptive tasks and the overall communications of results. According to some of the existing literature, descriptive tasks are usually the easiest in the writing of EIARs; therefore, such review areas are always performed relatively well [21,25]. Apart from the improvements seen in RA 1.0 (Descriptive tasks) and RA 5.0 (Communication of results), the quality of EIARs declined in several review areas. Furthermore, the frequency of instances with no improvements was relatively high, especially in the case of the following review areas:

- RA 2.0: Terrain susceptibility;
- RA 3.0: Impact identification and prediction;
- RA 4.0: Mitigation measures and alternatives.

Based on the results generated in the current study, the quality of selected EIARs is below the standard of EIA reporting documented in many international studies, although it is acknowledged that EIARs will always have problem areas, whether they are produced in the developed or developing countries. One of the implications that may be drawn from these results is that the sampled EIARs are not fit for the purpose they were supposed to fulfil; thus contributing negatively towards the attainment of sustainable development in the environmental management of mobile telecommunications infrastructure in Plateau State (Nigeria).

## 5. Conclusions and Recommendations 

This study has presented and discussed the different findings pertaining to the quality of EIARs regarding mobile telecommunication projects earmarked for construction in Plateau State. As Sandham et al. [20] maintains, conducting EIA processes in a successful manner depends, amongst other things, on the writing and generation of high-quality EIARs. Sound environmental decisions and the licensing of projects also depend on the quality of EIARs, thus explaining why there is a proliferation of studies that examine EIAR quality internationally [6,7,25,28,86]. 

The findings generated in the present study have addressed the different research objectives that were set out at the beginning of the research. Based on the various review areas, review categories, and review sub-categories in line with the adapted Lee and Colley Review Package, it has been indicated that the quality of the selected EIARs is grossly inferior and unsatisfactory (D-F) in many key areas. The quality was only satisfactory in the more descriptive tasks and the communication of results, thus depicting some aspects that may be regarded as areas of strengths. In contrast, many key environmental assessment tasks were either poorly attempted, inadequate, or entirely missing in those areas that required more analytical capabilities and adherence to international best practices on the part of the EAPs who compiled them. Areas of weaknesses entailed the identification and assessment of impacts, analysis of environmental base lines, estimation of terrain susceptibility, and the consideration of alternatives and formulation of mitigation measures. Such inferior EIAR quality outcomes mean that various environmental assessment tasks were not performed adequately, thus undermining the value of EIA as an impact prediction and mitigation planning instrument. Inevitably, the ‘fitness-for-purpose’ of the reviewed EIARs for aiding the decision-making process is considerably inadequate.

Furthermore, there was no marked improvement in the quality of EIARs between the two study periods (i.e., 2006–2012 and 2014–2015 periods), thus implying a serious lack of organizational learning and professional experience amongst the regulatory institutions in Nigeria and the environmental consulting companies that prepare and present EIARs to various stakeholders for joint environmental decision-making. Even more concerning from a sustainable development perspective is instances where the quality of EIARs actually deteriorated for the worse between the study periods. In the absence of satisfactory EIAR quality and chronological improvements over time, the reports produced for proposed mobile telecommunications projects are not only unhelpful for making sound environmental management decisions, but they undermine environmental stewardship and professional standards in the writing and compilation of EIARs.

As part of addressing these shortfalls, we recommend that EIARs in Nigeria should adhere to set EIA criteria and guidelines specified for the telecommunications infrastructure. There is also a need for more periodic evaluations of EIARs and further research in other economic sectors, the goal being to improve their legal and regulatory compliance and to build technical and methodological capacity for high-quality EIARs. Furthermore, the low quality of EIARs suggest the need for a renewed and robust professional certification body for the registration of professional environmental assessors in Nigeria. 

## Figures and Tables

**Table 1:** Some of the regulations guiding infrastructural developments in the telecommunications sector of Nigeria.

Guidelines for the review of EIA reports in Nigeria [47]
Harmful Waste Special Criminal Provisions (Act No. 42 of 1988) [51]
National Communication Commission Act No. 19 of 2003 (National Communication Commission of Nigeria 2003) [52]
National Environmental Standard and Regulations Enforcement Agency (Act No. 25 of 2007) [53]
Guidelines on Technical Specifications for the Installations of Masts and Towers (National Environmental Standards and Regulations Enforcement Agency 2009) [45]
National Environmental Standards for Telecommunications and Broadcast Facilities (National Environmental Standards and Regulations Enforcement Agency 2011) [46]

**Table 2:** Various grades of EIAR quality.

Symbol	Explanation	Quality Grade	Ranking
A	Relevant tasks well performed; no tasks conducted incomplete	A-B	Good (minor omissions)
B	Generally satisfactory and complete; only minor omissions and inadequacies	A-C	Satisfactory
C	Can be considered just satisfactory despite omissions and/or inadequacies	C	Borderline
D	Parts are well attempted but must, as a whole, be considered just unsatisfactory because of omissions or inadequacies	D-F	Poor (major omissions and inadequacies)
E	Not satisfactory, significant omissions or inadequacies		
F	Very unsatisfactory, important task(s) poorly performed or not attempted		
N/A	Not applicable. The review topic is not applicable, or it is irrelevant in the context of this report		

Source: Adapted from Lee et al. [74].

**Table 3:** Evaluation criteria used for the assessment of EIARs for the mobile telecommunication sector in Nigeria (* signifies the review areas and ^Ψ^ signifies the review categories).

Items.	Criteria Used for the Evaluation of EIAR Quality in the Present Study
1 *	Description of the project and environment
1.1 ^Ψ^	Planning
1.2 ^Ψ^	Spatial information relevant for the project area
1.3 ^Ψ^	Raw materials
1.4 ^Ψ^	Site construction activities of base station
1.5 ^Ψ^	Site selection considerations
1.6 ^Ψ^	Environmental baseline data
1.7 ^Ψ^	Description of project sites
1.8 ^Ψ^	Potentials of the area
2 *	Description of existing environment and environmental site selection considerations
2.1 ^Ψ^	Potential conflict with land uses
2.2 ^Ψ^	Significant environmental problems in the project sites
3 *	Terrain susceptibility for the proposed project area
3.1 ^Ψ^	Terrestrial habitat alterations
3.2 ^Ψ^	Avian collisions
3.3 ^Ψ^	Aquatic habitat alterations
3.4 ^Ψ^	Visual impacts
3.5 ^Ψ^	Impact prediction
3.6 ^Ψ^	Scoping
4 *	Mitigation measures/alternatives
4.1 ^Ψ^	Measures to reduce or avoid animals in all phases of the project
4.2 ^Ψ^	Procedures to minimize avian collisions
4.3 ^Ψ^	Measures to prevent and control visual impacts
4.4 ^Ψ^	Hazardous materials management action
4.5 ^Ψ^	Measures to minimize EMF radiation on biodiversity and man
5 *	Communication of results
5.1 ^Ψ^	Layout
5.2 ^Ψ^	Presentation
5.3 ^Ψ^	Emphasis
5.4 ^Ψ^	Non-technical summary

**Table 4:** EIARs selected for the assessment of quality.

No.	Project Type	EIARs Year of Submission	Total No. of EIARs Submitted	No. of EIARs Sampled for the Study	Percentage (%) of EIAR Sampled for the Study
1	Telecommunication BTSs	2006	24	20	83%
2	Telecommunication BTSs	2012	46	20	43%
3	Telecommunication BTSs	2014	177	20	11%
4	Telecommunication BTSs	2015	41	20	51%

**Table 5:** Allocation of quality to environmental assessment tasks performed under Review Area 1.

Summary of Review Category Grades	A	B	C	D	E	F	A-C%	D-F%	A-B%	C-D%	E-F%
Planning	28	32	11	4	5	0	89	11	75	19	6
Spatial information covering the project	18	22	16	9	3	12	70	30	50	31	19
Raw material	8	30	2	25	10	5	50	50	48	34	18
Site construction activities of base station	21	15	24	12	5	3	75	25	45	45	10
Site selection consideration	23	18	21	6	12	0	78	22	51	34	15
Baseline data	8	30	24	10	5	3	78	22	48	42	10
Description of the prosed project sites	0	5	23	18	22	12	35	65	6	51	43
Potentials of the area	2	9	5	4	22	38	20	80	14	11	75

**Table 6:** Allocation of quality to environmental assessment tasks performed under Review Area 2.

	Summary of Review Category Grades	A	B	C	D	E	F	A-C%	D-F%	A-B%	C-D%	E-F%
2.1	Potential conflict with land uses	0	0	0	0	0	80	0	100	0	0	100
2.2	Significant environmental problems in project sites	3	4	13	4	24	32	25	75	9	21	70

**Table 7:** Allocation of quality to environmental assessment tasks performed under Review Area 3.

	Summary of Review Category Grades	A	B	C	D	E	F	A-C%	D-F%	A-B%	C-D%	E-F%
3.1	Terrestrial habitat alteration	0	0	0	16	10	54	0	100	0	20	80
3.2	Avian collision	0	0	0	18	6	56	0	100	0	22	73
3.3	Aquatic habitat alteration	0	0	0	0	0	80	0	100	0	0	100
3.4	Visual impacts	0	0	0	0	0	80	0	100	0	0	100
3.5	Impact prediction	9	28	23	8	5	7	75	25	46	39	15
3.6	Scoping	0	0	0	0	0	80	0	100	0	0	100

**Table 8:** Allocation of quality to environmental assessment tasks performed under Review Area 4.

	Summary of Review Category Grades	A	B	C	D	E	F	A-C%	D-F%	A-B%	C-D%	E-F%
4.1	Measures to reduce or avoid animals in all phases of the project	0	0	0	0	0	80	0	100	0	0	100
4.2	Procedures to minimize avian collision	0	0	0	0	0	80	0	100	0	0	100
4.3	Measures to prevent and control visual impacts	0	0	0	0	0	80	0	100	0	0	100
4.4	Hazardous materials management action	0	0	0	0	0	80	0	100	0	0	100
4.5	Measures to minimize EMF radiation on biodiversity and man	0	0	0	0	0	80	0	100	0	0	100

**Table 9:** Allocation of quality to environmental assessment tasks performed under Review Area 5.

Summary of Review Category Grades	A	B	C	D	E	F	A-C%	D-F %	A-B %	C-D %	E-F%
5.1 Layout	11	23	32	10	3	1	83	17	43	52	5
5.2 Presentation	12	27	35	1	3	2	92	8	49	45	6
5.3 Emphasis	0	17	50	6	5	2	83	17	21	70	9
5.4 Non-technical summary	0	0	0	0	0	80	0	100	0	0	100

**Table 10:** Areas of strengths (A-B) in the EIARs assessed for their quality.

Review Categories (Strengths)	Proportion of EIARs with > 50% (A-B) Symbols
1.1 Planning	75%
1.5 Site selection considerations	51%

**Table 11:** Areas of weaknesses (E-F) in the EIARs assessed for their quality.

Review Categories (Weaknesses)	Proportion of EIARs with >50 (E-F) Symbols
2.1 Potential conflict with land uses	100%
3.3 Aquatic habitat alterations	100%
3.4 Visual impacts	100%
3.5 Scoping	100%
4.1 Measures to reduce or avoid animals in all project phases	100%
4.2 Procedures minimizing avian collisions	100%
4.3 Measures to prevent and control visual impacts	100%
4.4 Hazardous materials management action	100%
4.5 Measures to minimize EMF radiation on biodiversity and man	100%
5.4 Non-technical summaries	100%
1.8 Potentials of the area	75%
3.2 Avian collisions	73%
2.2 Significant environmental problems in the project sites	70%

**Table 12:** Degree of changes in the quality of EIARs between Period 1 and Period 2.

Review Areas (RAs) and Review Categories (RCs)		(Period 1) 2006–2012 EIARs			(Period 2) 2014–2015 EIARs		
		A-C%	A-B%	E-F%	A-C%	A-B%	E-F%
RA 1.0	Description of the project and environment	62.5	25%	0	92.5	47.5	5%
1.1	Planning	87.5%	72.5.%	7.5%	90%	77.5%	5%
1.2	Spatial information	65%	50%	22.5%	75%	50%	15%
1.3	Raw materials	50%	47.5%	20%	50%	47.5%	17.5%
1.4	Site constructions	75%	45%	7.5%	75%	45%	12.5%
1.5	Site selections	77.5%	52.5%	15%	77.5%	50%	15%
1.6	Baseline data	77.5%	47.5%	10%	77.5%	47.5%	10%
1.7	Description of project sites	35%	5%	42.5%	35%	7.5%	42.5%
1.8	Land potential	25%	15%	70%	15%	12.5%	80%
RA 2.0	Terrain susceptibility	0	0	100%	0	0	100%
2.1	Potential conflict with land uses and suitability	0	0	100%	0	0	100%
2.2	Significant environmental problems in the project sites	100	100%	100%	100%	100%	100.%
3.0	Impact identification and prediction	0	0	100%	0	0	100%
RA 3.1	Terrestrial habitat alteration	0	0	100%	0	0	100%
3.2	Avian collision	0	0	100%	0	0	100%
3.3	Aquatic habitat alteration	0	0	100%	0	0	100%
3.4	Visual impacts	0	0	100%	0	0	100%
3.5	Impact prediction	73%	45%	18%	78%	48%	13%
3.6	Scoping	0	0	100%	0	0	100%
RA 4.0	Mitigation/Alternatives	0	0	100%	0	0	100%
4.1	Measures to reduce or avoid animals in all phases of the project	0	0	100%	0	0	100%
4.2	Procedures to minimize avian collision	0	0	100%	0	0	100%
4.3	Measures to prevent and control visual impacts	0	0	100%	0	0	100%
4.4	Hazardous materials management action	0	0	100%	0	0	100%
4.5	Measures to minimize EMF radiation on biodiversity and man	0	0	100%	0	0	100%
4.6	Alternatives	0	0	100%	0	0	100%
RA 5.0	Communication of results	63%	17%	17%	65%	17%	17%
5.1	Layout	80%	40%	7.5%	85%	45%	2.5%
5.2	Presentation	92.5%	47.5%	7.5%	92.5%	50%	5%
5.3	Emphasis	82.5%	20%	10%	85%	22.5%	7.5%
5.4	Non-technical summary	0	0	100	0	0	100%

Key/Legend: Quality of EIARs, 
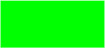
: Improvement in EIAR Quality, 
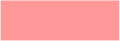
:Decline in EIAR quality, 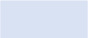: No change in EIAR quality.

## Data Availability

Not applicable.
